# Forecasting outpatient visits using empirical mode decomposition coupled with back-propagation artificial neural networks optimized by particle swarm optimization

**DOI:** 10.1371/journal.pone.0172539

**Published:** 2017-02-21

**Authors:** Daizheng Huang, Zhihui Wu

**Affiliations:** Department of Biomedical Engineering, School of Preclinical Medicine, Guangxi Medical University, Nanning, Guangxi Province, China; Southwest University, CHINA

## Abstract

Accurately predicting the trend of outpatient visits by mathematical modeling can help policy makers manage hospitals effectively, reasonably organize schedules for human resources and finances, and appropriately distribute hospital material resources. In this study, a hybrid method based on empirical mode decomposition and back-propagation artificial neural networks optimized by particle swarm optimization is developed to forecast outpatient visits on the basis of monthly numbers. The data outpatient visits are retrieved from January 2005 to December 2013 and first obtained as the original time series. Second, the original time series is decomposed into a finite and often small number of intrinsic mode functions by the empirical mode decomposition technique. Third, a three-layer back-propagation artificial neural network is constructed to forecast each intrinsic mode functions. To improve network performance and avoid falling into a local minimum, particle swarm optimization is employed to optimize the weights and thresholds of back-propagation artificial neural networks. Finally, the superposition of forecasting results of the intrinsic mode functions is regarded as the ultimate forecasting value. Simulation indicates that the proposed method attains a better performance index than the other four methods.

## Introduction

Obtaining healthcare in China is currently challenging because the growth rate of healthcare agencies is far lower than the rise in patient needs. Accurately forecasting the number of outpatient visits will increase the efficiency of planning and the delivery of outpatient management. This ability can also help healthcare administrators oversee hospitals effectively, reasonably organize schedules for human resources and finances, and properly distribute hospital material resources. Therefore, forecasting the number of outpatient visits has become an important issue in public health and has motivated many researchers to establish mathematical models to realize such predictions, especially in China. Ching proposed a fuzzy time series method based on the weighted-transitional matrix, as well as the expectation method and grade-selection approach, to forecast the number of outpatient visits[1]. Meanwhile, Esmaeil Hadavandia presented a hybrid artificial intelligence model for the development of a Mamdani-type fuzzy-rule-based system to forecast outpatient visits[2]. Decomposition and multi-local predictor fusion were proposed to predict outpatient consults for diarrhea[3].

The number of outpatient visits is a nonlinear and nonstationary time series. The forecast of this information may not be perfectly accurate if the linear method is used in such time series. Meanwhile, empirical mode decomposition(EMD) is an empirical, intuitive, direct, and self-adaptive time–frequency data analysis method introduced by Huang et al.[4]. This approach is fairly versatile in a broad range of applications on signal extraction from data generated in noisy nonlinear and nonstationary processes. Compared with Fourier transformation and wavelet transformation, it has many advantages such as good multi-resolution and wide applicability[5]. EMD technique has been successfully applied in various areas, such as signal processing[6], digital holography[7], image processing[8], detection techniques[9], and forecasting approach[10, 11]. The original morbidity data of outpatient visits can be decomposed into several sub-series by EMD. Compared with the original series, the sub-series exhibits a more apparent regularity and can be forecasted to achieve easy prediction tasks and fine results.

Though the original morbidity data of outpatient visits has been decomposed into several sub-series, the sub-series are still time series. There were many applications using the traditional statistical models like autoregressive model, moving average model and autoregressive moving average models for predicting time series in past studies. These models perform well when the data lie within the range of past observations. But they perform poorly to predict extremes and also when the data are lying just near to limits[12]. The application of artificial intelligence prediction models has been offered in recent years. One of the most widely used artificial intelligence methods in prediction is artificial neural networks (ANNs) [13]. ANNs are complex and flexible nonlinear systems inspired by biological neural networks (e.g., animal central nervous systems, particularly, the brain) and are used to estimate or approximate functions, which can depend on numerous inputs and are generally unknown[14,15]. ANN processes information by adjusting the internal relations between large connected nodes and it has strong self-learning abilities and adaptive abilities [16].The ANNs time series models can capture the historical information by nonlinear functions [17]. A highly popular and widely used ANN algorithm is the back-propagation ANN (BPANN) [18], which involves connection weights and thresholds adjusted by the backward propagation of errors. One major application area of BPANN is in forecasting, owing to its characteristics of extreme computational power, massive parallelism, and fault tolerance [19].

Network performance is affected by the initial weight and threshold of BPANN, and these parameters participate in a close relationship with the network convergence, falling into the local minimum and training time. To overcome the problem, different optimization algorithms optimize the initial weight and threshold of BPANN, including genetic algorithm [20,21], ant colony algorithm[22], simulated annealing algorithm[23] and particle swarm optimization(PSO). PSO is a type of swarm intelligence optimization algorithm for global optimization and has proven to be a competitor to GA when it comes to optimization problems[24].Compared with other biological evolution algorithms, PSO occupies the bigger optimization ability using simple relations [25]. It is widely used in optimization because of its need for less parameter sets and its faster convergence rate and easy escape from the local optimum compared with other algorithms[26–31]. At the same time, it can perform strong parallel search and global optimization. PSO was thus selected to optimize the BPANN weights and thresholds in the present study because of its simplicity and good performance in finding desirable solutions.

This study aims to assess the forecasting accuracy of BPANN models coupled with EMD for outpatient visits. EMD is applied to decompose the original data of outpatient visits, and then different BPANN models are constructed with each sub-series. The final forecasted value is obtained by summing the forecasted value of each sub-series.

## Methods

### Empirical mode decomposition

EMD is an adaptive and efficient method to analyze nonlinear and non-stationary signals. Without any a priori knowledge, the original time series was decomposed into a sum of oscillatory functions called intrinsic mode functions (IMFs)[4,32,33]. The IMFs must meet the following conditions:

1. The number of extrema (maximum and minimum) and the number of zero-crossings must be the same or differ at most by one in the whole data set.
2. At any point, the IMF is symmetric with respect to the local zero mean.

The IMF can be extracted from the original time series through a shifting process described as follows:

*Step 1*. All the local extrema [maximum *e*_*max*_(*t*) and minimum *e*_*min*_(*t*)] points of the given time series *x*(*t*) are calculated.
*Step 2*. All the local maxima points are connected as the upper envelop, and all the minima points as the lower envelop, by a cubic spline line.
*Step 3*. The mean *m*(*t*) of the upper and lower envelops are calculated as
  $$m\;(t)=(e_{\max}(t)+e_{\min}(t))/2$$ (1)
*Step 4*. The mean from the original time series is obtained, and the difference is defined as *h*(*t*):
  h(t)=x(t)−m(t) (2)
*Step 5*. *h*(*t*) is checked and judged whether it meets the two conditions for an IMF in accordance with the stopping criterion. If the criteria are satisfied, then *h*(*t*) is denoted as the first IMF and written as *g*_*1*_(*t*) = *h*(*t*). Moreover, *x*(*t*) is replaced with the residue *r*(*t*) *= x*(*t*)*-h*(*t*). If the conditions are not satisfied, *x*(*t*) is replaced with *h*(*t*) and steps 1 to 4 are repeated until *h*(*t*) meets the two conditions for an IMF.
*Step 6*. Steps 1 to 5 are repeated. The shifting procedure is then terminated until the residue becomes a constant, a monotonic function, or a function with only one maximum and one minimum from which no more IMF can be extracted. *g*_*i*_(*t*)+*r*_*m*_(*t*)

Then, the original time series *x*(*t*) is expressed as the sum of the IMFs and a residue:

$$x(t)=\sum_{i=1}^{m}g_{i}(t)+r_{m}(t)$$ (3)

where *m* is the number of IMFs and *r*_*m*_(*t*) is the final residue.

### Back-propagation artificial neural networks

ANN approach can imitate any complex and non-linear relationship through non-linear units (neurons) and has been widely used in the forecasting area. BPANN is the most extensively used ANN model.[5,34,35]. The typical topology of BPANN involves three layers: input layer, where the data are introduced to the network; hidden layer, where the data are processed; and output layer, where the results of the given input are produced[15]. A number of interconnected neuron nodes are present in the layers. The output *H*_*j*_ of any neuron can be represented as

$$H_{j}=f(\sum_{i=1}^{n}\omega_{ij}x_{i}-b_{j})$$ (4)

where *n* is the number of inputs, *x*_*i*_ is the *i*th input, *ω*_*ij*_ is the connective weight between the *j*th and *i*th neurons, *b*_*j*_ is the threshold, *f* is a nonlinear activation function, and the sigmoid function *f*(*t*) = 1/(1+*e*^*-x*^) is widely employed as the activation function. Determining the optimum weights and thresholds is usually performed by trial during the training stage as the output of the network matches the desired pattern for a specific set of inputs.

The algorithm includes training and forecast, which are detailed in the following steps:

*Step 1*. Forward propagation of input signal. The input vector propagates to the output layer after being computed by the hidden layer.
*Step 2*. Backward propagation of error signal. The error propagates backward through the original neural network if the error value does not meet the given tolerance.
*Step 3*. Weight and threshold updates. The weights and thresholds are adjusted by the backward propagation of errors until the error value meets the given tolerance. Then, the fixed structure of the BPANN model is obtained.
*Step 4*. Forecast. The trained BPANN model is used for forecast.

### Particle swarm optimization

PSO is a type of swarm intelligence optimization algorithm for global optimization proposed by Eberhart and Kennedy in 1995 and inspired by the behavior of a swarm of birds [36].The social behavior of birds is simulated in PSO. Each bird is represented by a particle, and a collection of particles is identified as a swarm. Each particle in the swarm represents a potential solution to the optimization problem. Particles are simultaneously updated by exchanging information with one another. The basic PSO algorithm is governed by Eqs (5) and (6):

$$V_{i}^{k+1}=\omega V_{i}^{k}+c_{1}r_{1}(P_{i}^{k}-X_{i}^{k})+c_{2}r_{2}(P_{g}^{k}-X_{i}^{k})$$ (5)

$$X_{i}^{k+1}=X_{i}^{k}+V_{i}^{k+1}$$ (6)

where *V*_*i*_ represents the velocity of the *i*th particle. *X*_*i*_ represents the position of the *i*th particle and denotes a potential solution to the problem. *P*_*i*_ is the best solution obtained by particle *i* until iteration *k*, and *P*_*g*_ is the best solution obtained by all particles until iteration *k*. *ω*∈[0, 1] is the inertia weight. *r*_1_ and *r*_2_ are independent random numbers uniformly distributed in the range of [0, 1], whereas *c*_1_ and *c*_2_ are positive constants that are both acceleration factors, named cognitive and social parameters respectively. *k* represents the current iteration of the optimization process.

The speed and precision of convergence are greatly affected by the initial parameters in PSO. Therefore, selecting the reasonable the initial parameters in the algorithm is important.

1. Selecting the inertia weight. Inertia weight is the most important parameter in the PSO, and the use of an appropriate inertia weight can help achieve the balance between global search and local optimization. The global search ability of the algorithm is enhanced when the inertia weight is large. By contrast, the local search performance becomes effective and enables the search for the global optimal solution when the inertia weight is relatively small. The linear decreasing inertia weight strategy was proposed by Y. Shi in 1999[37]. The ability of global and local searches is balanced in this method, which accelerates the convergence rate of the algorithm. The linear decreasing inertia weight is calculated as follows:

$$\omega =\omega_{start}-\frac{\omega_{start}-\omega_{end}}{t_{\max}}\times t$$ (7)

where *ω*_*start*_ and *ω*_*end*_ are the initial and termination inertia weights respectively. The typical inertia weight is adapted from 0.9 to 0.3. *t*_*max*_ is the maximum iteration, and *t* is the current iteration. The linear decreasing inertia weight was used in this work.

2. Selecting the learning factors. The learning factors reflect the exchange information between particles. These aspects are in the range of [1, 2.5] by subjective experience.

The adaptive time-varying strategy for adjusting the learning factor was proposed by A. Ratnawecra in 2004[38]. The adjustment formula is as follows:

$$c_{1}=c_{1start}+\frac{c_{1end}-c_{1start}}{t_{\max}}\times t$$ (8)

$$c_{2}=c_{2start}+\frac{c_{2end}-c_{2start}}{t_{\max}}\times t$$ (9)

The typical number of the learning factor is that the *c*_1_ reduces from 2.5 to 0.5, whereas the *c*_2_ increases from 0.5 to 2.5. Results show that the performance is enhanced by the adaptive time-varying strategy than by the fixed learning factor. Thus, the adaptive time-varying method was used in this study.

The thresholds and weights of BPANN are encoded as a particle. The dimension *D* of particle swarm is as follows:

$$D=n_{h}+n_{0}+n_{i}\times n_{h}+n_{h}\times n_{0}$$ (10)

where *n*_*i*_, *n*_*h*_, and *n*_*0*_ are the input, hidden, and output nodes, respectively.

The mean square error (MSE) between the prediction and raw data is considered as the fitness function used to evaluate the quality of particles.

The velocity and position of these particles with higher fitness are updated until the best particles are produced.

**Fig 1:**
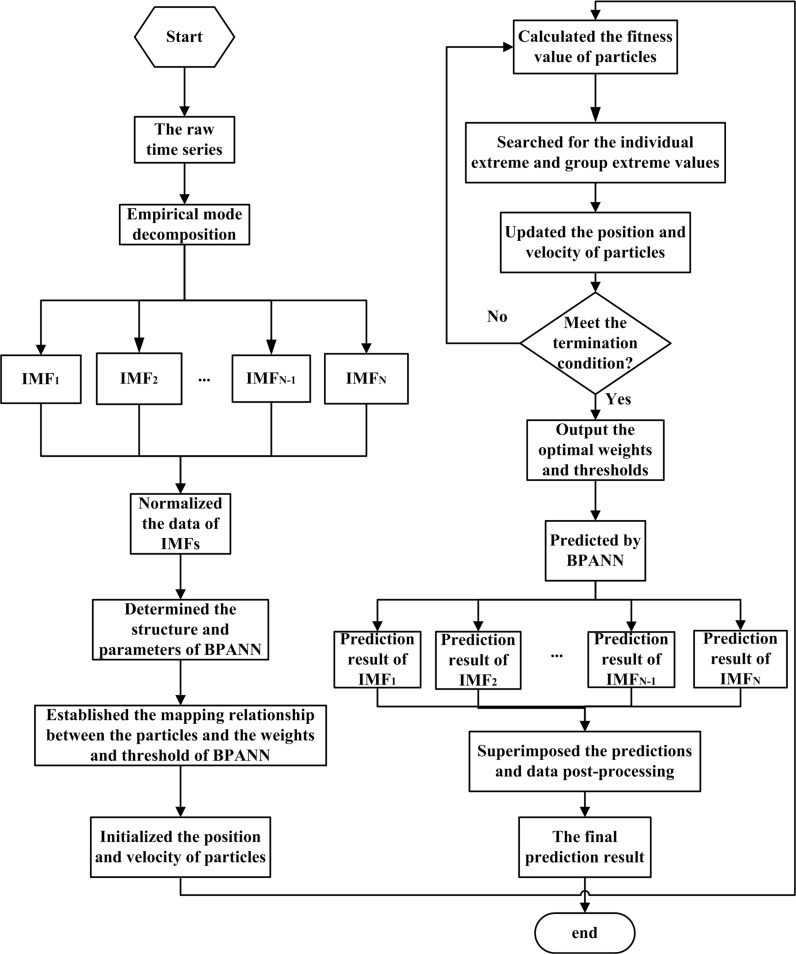
Flow chart of the proposed algorithm.

The flow chart of the proposed method in the paper is shown in Fig 1.

The proposed algorithm can be separated into six steps. (1) Raw time series decomposition (Fig 1). The number of outpatient visits is released from the hospital information section by month and considered as the original time series. Then, EMD is used to decompose the raw time series into a finite and often small number of IMFs plus a residue. Each IMF can represent the local characteristic time scale by itself. (2) Data normalization. By directly calling the two functions premnmx and postmnmx in MATLAB software, each IMF is normalized by the Min-Max normalization algorithm. (3) Thresholds and weights of BPANN optimization. The weights and thresholds of BPANN are mapped to and encoded as the particles. The optimal particles are found through updates of their positions and velocities. (4) BPANN training. The optimal weights and thresholds are employed as the initial weights and thresholds and adopted into the BPANN. The training data are also considered into the network. The prediction network can be obtained through training. (5) Trained BPANN testing. Each prediction result of the IMFs can be obtained by using the trained BPANN. (6) Superposition of the IMF forecast results. Finally, the superposition of forecasting results of the IMFs is regarded as the ultimate forecasting value.

## Results and discussion

### Data sources

A case will be simulated in this section. The number of outpatient visits from a hospitals in Nanning City, China was documented (S1 Dataset).

### Ethical review

The study protocol and utilization of outpatient visit data were reviewed from a hospitals in Nanning City, China, and no ethical issue was identified. Therefore, no ethical approval was required.

**Fig 2:**
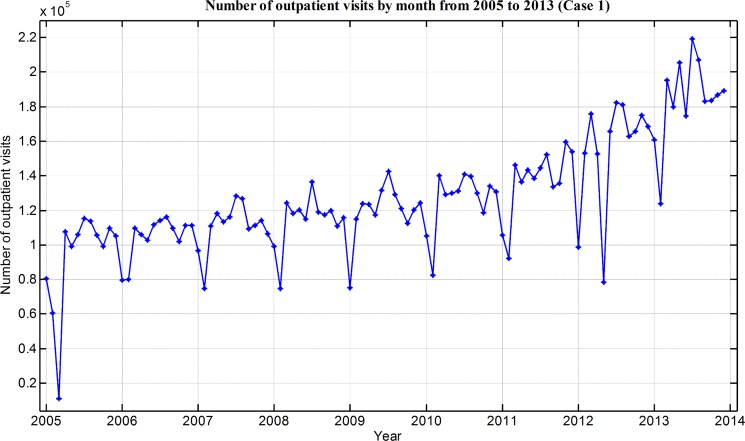
Number of outpatient visits by month from 2005 to 2013.

The number of outpatient visits from January 2005 to December 2013 are obtained as the original time series and shown in Fig 2.

**Fig 3:**
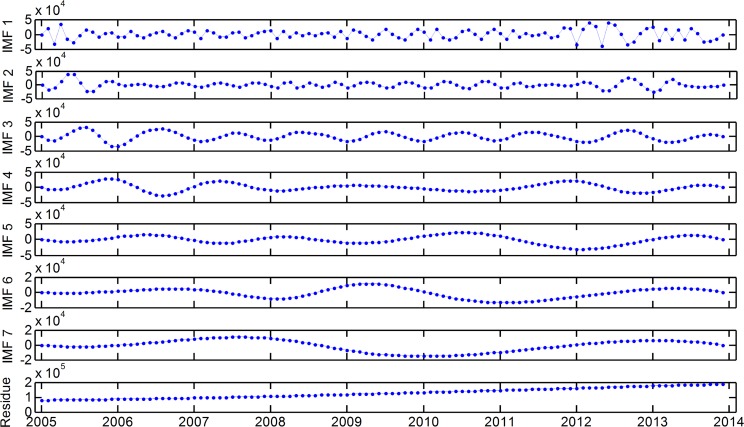
Decomposition of outpatient visits.

The decomposition results obtained through the EMD technique are shown in Fig 3. Seven IMFs and a residue component are observed. The IMFs present the characteristics of outpatient visit fluctuations on different time scales from high frequency to low frequency. IMF1 involves an obvious periodic variability and exhibits the maximum amplitude, highest frequency, and shortest wavelength. The following IMF components decrease in amplitude and frequency and increase in wavelength. The last residue is a mode that slowly varies around the long-term average; this mode shows the overall trend of outpatient visits.

**Fig 4:**
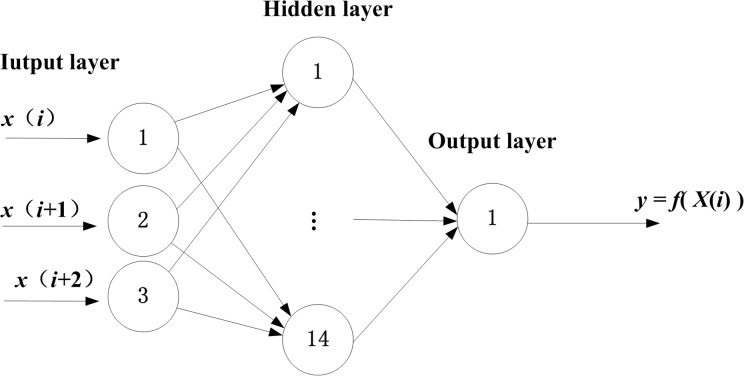
BPANN model.

There is no mature reference method to choose appropriate network structure and neuron for building a BPANN. The optimal three input layer neurons were experimentally selected and have average square error less than 0.01. The output layer only contains one neuron representing the forecast value. Some empirical formulas are used to choose hidden layer node number. Such as $n_{2}=\sqrt{n_{1}+n_{3}}+m$, where *n*_1_, *n*_2_, *n*_3_ and *m* represent the input node, hidden node, output node and a positive integer and *m*∈[1,10]. After computing, the prediction is found to be most accurate when *m* = 10 instead of other values. Therefore, a three-layer proposed model with 3 input nodes, 14 hidden nodes, and 1 output node (3–14–1) is obtained (Fig 4). The selection for parameters of BPNN is based on the literature [39]. And the parameters of BPANN are listed in Table 1.

**Table 1:** Parameters of BPANN.

Parameter	Value	Parameter	Value	Parameter	Value
Input node	3	activation function of output layer	purelin	Net.tralinParam.show	25
Hidden node	14	training function	trainrp	Net.tralinParam.goal	0.01
Output node	1	learning function	learngdm	Net.tralinParam.lr	0.15
Activation function of the hidden layer	tansig	Net.tralinParam.epochs	1000	Net.tralinParam.min_grad	1e-6

In order to optimize the weights and thresholds of the BPANN mode, PSO is employed. Four important parameters of the PSO algorithm should be set: inertia weight, learning factors, the maximum number of iterations, and the dimensions of particle swarm. The linear decreasing inertia weight was used in this work just as previously introduced. As originally developed, the inertia weight often decreases linearly from about 0.9 to 0.4 during a run [40,41]. Here we set it from about 0.9 to 0.3. And the adaptive time-varying method was used in this study for the performance is enhanced by the adaptive time-varying strategy than by the fixed learning factor. The thresholds and weights of BPANN are encoded as a particle, 71 of dimensions will be attained based on the Eq (10). The number of iterative learning was 200. The parameters of PSO are listed in Table 2.

**Table 2:** Parameters of PSO.

Parameter	Value	Parameter	Value	Parameter	Value
Population	40	minimum fitness	1e-30	iteration	200
Dimension	71	maximum velocity	1	minimum velocity	−1
Inertia weight	linear decrease	maximum weight	0.9	minimum weight	0.3
Learning factor	adaptive time-varying strategy	learning factor C_1_	0.5−2.5	learning factor C_2_	0.5−2.5

The raw time series obtained from January 2005 to November 2012 are adopted as training samples, and those obtained from December 2012 to December 2013 are employed as testing samples.

**Fig 5:**
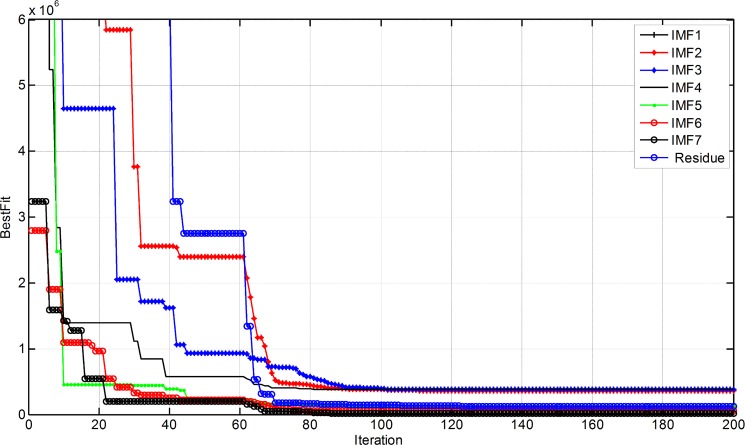
Variation of the best fitness with iteration.

The weights and thresholds of BPANN are optimized by PSO by training each component. The variation of the best fitness with iteration is illustrated in Fig 5.

**Fig 6:**
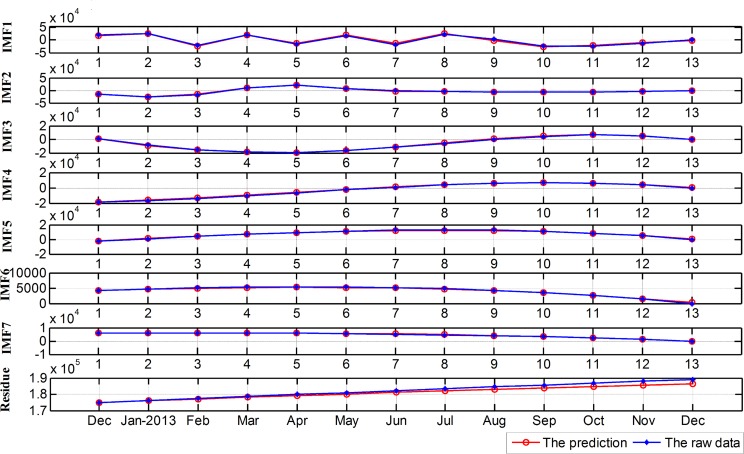
Result prediction and raw data of each component from December 2012 to December 2013.

The result prediction and the raw data of each component are shown in Fig 6.

**Fig 7:**
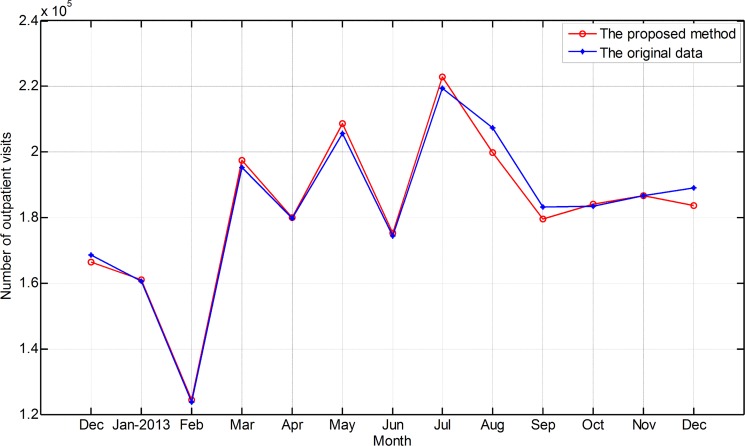
Prediction value and the observed data.

The ultimate forecasting value and the observed data are illustrated in Fig 7.

To compare the result prediction with other methods, a simulation is performed by PSO-BPANN directly, GA-BPANN directly, wavelet decomposition PSO-BPANN(WD-PSO-BPANN), wavelet decomposition GA-BPANN(WD-GA-ANN), and the proposed methods(abbreviated as EMD-PSO-BPANN). These methods hold the same simulation conditions.

Four main criteria are used for evaluation of level prediction and directional forecasting including the coefficient of correlation (R), root mean squared error (RMSE), mean average percentage error (MAPE), and sum of squared error (SSE). The definitions are shown as follows.

R criterion is given by

$$R=\frac{R_{\hat{x}x}}{std(x)std(\hat{x})}$$ (11)

RMSE criterion is defined as

$$RMSE=\sqrt{\frac{1}{n}{\sum_{i=1}^{n}\left({\hat{x}}_{i}-x_{i}\right)}^{2}}$$ (12)

MAPE criterion is calculated through

$$MAPE(\%)=\frac{1}{n}\sum_{i=1}^{n}\left|{\hat{x}}_{i}-x_{i}\right|*100$$ (13)

The SSE criterion is given by

$$SSE={\sum_{i=1}^{n}\left({\hat{x}}_{i}-x_{i}\right)}^{2}$$ (14)

where ${\hat{x}}_{i}$ is the forecasted data and *x*_*i*_ is the actual data. ${\bar{x}}_{i}$ denotes the actual data’s mean. *n* is the number data points considered.

**Fig 8:**
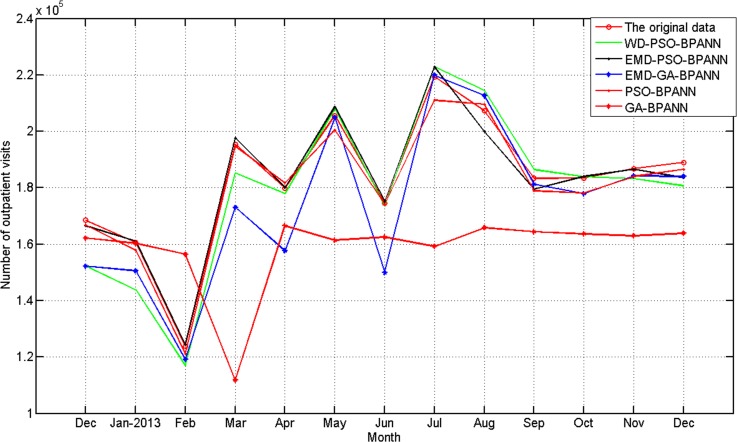
Comparison predictions with five methods from December 2012 to December 2013.

The result predictions by the five methods are shown in Fig 8.

The comparison of evaluation indexes are listed in Table 3. We noted that the RMSE can provide a good measure of model performance for high flows[42] and was selected as the performance criterion of level prediction. MAPE is a relative criterion which is sensitive to the forecasting errors that occur in the low(er) magnitudes of each dataset. SSE is a measure of the discrepancy between the data and an estimation model. A small SSE indicates a tight fit of the model to the data. These three performance indexes are smaller, and the prediction result is more accurate. R has been widely used for model evaluation and was selected as the degree of collinearity criterion of level prediction [41]. The correlation coefficient is larger, and the correlation between the predicted and the observed data is improved. It can been seen from Tabel.8, the proposed method holds advantages over the other four methods in all the evaluation indexes except the indexes of R, thus proving the effectiveness of the prediction by the proposed approach.

**Table 3:** Comparison of evaluation indexes (best performers are in bold font).

Methods	Index Performance			
	R	RMSE	MAPE	SSE
WD-PSO-BPANN	0.9759	8.1564e+3	0.13	8.6485e+8
EMD-PSO-BPANN	0.9910	**3.1653e+3**	**0.0241**	**1.3024e+8**
EMD-GA-BPANN	0.9442	12.661e+3	0.34	208.38e+8
PSO–BPANN	**0.9925**	3.7786e+3	0.11	1.8561e+8
GA–BPANN	-0.0687	36.824e+3	1.13	176.28e+8

The relationship between the seasonal fluctuation index of outpatient visits and the five models prediction results is described in the followings [43].

The seasonal fluctuation index of the same month in eleven years from 2005 to 2013 can be calculated as:

$$SFI1=\frac{\left|{\bar{x}}_{same}-{\bar{x}}_{all}\right|}{{\bar{x}}_{all}}$$ (15)

where ${\bar{x}}_{same}$ is the average outpatient visits of the same month and ${\bar{x}}_{all}$ is the average outpatient visits of all of the months from 2005 to 2013. Obviously, *SFI*1 indicates seasonal characteristics of the outpatient visits.

The seasonal fluctuation index of the every month from December 2012 to December 2013 is calculated as:

$$SFI2=\frac{\left|x_{i}-\bar{x}\right|}{\bar{x}},i=1,\ldots ,13$$ (16)

Where *x*_*i*_ is the outpatient visits in each month and $\bar{x}$ is the average outpatient visits of all of the months from December 2012 to December 2013.

The relative error of prediction is used to measure and defined as:

$$RE_{i}=\frac{\left|{\hat{x}}_{i}-x_{i}\right|}{x_{i}},i=1,2\ldots ,n$$ (17)

where ${\hat{x}}_{i}$ is the predicted value and *x*_*i*_ the observed values.

**Fig 9:**
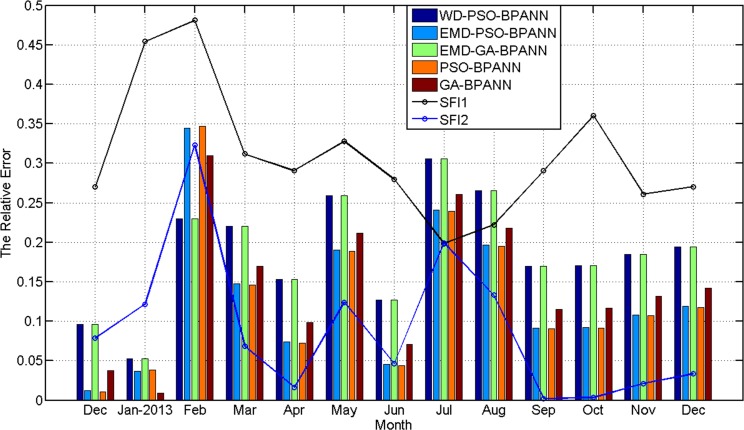
The relationship between the seasonal fluctuation index and RE of the predictions by the five methods. (Histograms and curves represent RE of the predictions and the seasonal fluctuation index, respectively).

The relative error of the five prediction results and the seasonal fluctuation index of outpatient visits are shown in Fig 9.

It can be seen from Fig 8 that: 1) the curve of *SFI*1 indicates the outpatient visits has obvious seasonal characteristics. The outpatient visits which happens annually in January and February is relatively high with a rapid decline in July. Other months are relatively stable; 2) the greater the seasonal fluctuation index of the every month from December 2012 to December 2013, the greater the relative error of the five methods. and 3) the absolute error of the WD-PSO-BPNN and EMD-GA-BPANN are larger than that of the other three methods when the outpatient visits data is stable, such as from September to October; the absolute error of the EMD-PSO-BPANN and PSO-BPANN are smaller than that of the other three methods in all the prediction months.

## Conclusion

A new forecasting method that combines EMD and BPANNs based on PSO is proposed to forecast outpatient visits. Simulation results show that this method can improve forecasting and thus help policy makers manage hospitals effectively.

## Supporting information

S1 Dataset Number of outpatient visits by month from 2005 to 2013. (XLS)
